# Why does melanoma metastasize into the brain? Genes with pleiotropic effects might be the key

**DOI:** 10.3389/fgene.2013.00075

**Published:** 2013-05-01

**Authors:** Anatoliy I. Yashin, Deqing Wu, Konstantin G. Arbeev, Alexander M. Kulminski, Eric Stallard, Svetlana V. Ukraintseva

**Affiliations:** Center for Population Health and Aging, Social Science Research Institute, Duke UniversityDurham, NC, USA

## Introduction

Melanoma is the most aggressive type of skin cancer. It is the seventh most common type of cancer among men and the eighth most common among women with a lifetime risk about 2% (Feng et al., 2011). The incidence of melanoma is rising faster than that of any other cancer type in the US (Tsao et al., 2004). Melanoma is a multifactorial disease whose risk depends on genetic susceptibility (around 10% of melanoma cases have a family history of the disease) as well as on external factors, among which an exposure to ultraviolet (UV) radiation and sunburn play an important role.

Brain metastases is a major challenge in melanoma and one of the least understood aspects of this disorder (Skibber et al., 1996; Fidler et al., 1999). Average survival in advanced metastatic melanoma is only 6–10 months with <5% of patients living 5 years after diagnosis (Jemal et al., 2002). More than half of all melanoma deaths occur from brain metastasis. A key event in brain metastasis is the migration of cancer cells through the blood brain barrier (BBB) (Arshad et al., 2010). The BBB is formed by specialized endothelial cells lining capillaries in the central nervous system. Brain capillary walls are more difficult to penetrate due to a tight layer of endothelial cells, tight junctions (TJs), and other structures that restrict the diffusion of microscopic objects (e.g., bacteria) and large or hydrophilic molecules into the cerebrospinal fluid.

In order to allow the melanoma cell to metastasize into the brain, the integrity of the BBB has to be compromised. This suggests that some germ line mutations contributing to metastatic melanoma may also increase the permeability of the BBB. Finding such mutations and understanding the mechanisms of their action could make substantial contributions to reducing mortality from melanoma. Currently, very little is known about the molecular mechanisms by which melanoma cells can penetrate the BBB.

The literature on germ line mutations contributing to melanoma and its metastases provides some clues about relevant genes and their functions. Udart et al. (2001) gave evidence that a number of genes which are likely to play a role in melanoma and metastases are located on chromosome 7. The list includes the EGFR gene encoding the epidermal growth factor receptor; the BRAF gene, which is a member of the Raf kinase family of serine/threonine-specific protein kinases involved in the MAP kinase/ERKs signaling implicated in many cancers (Wangari-Talbot and Chen, 2012); the PDGF-A gene encoding for platelet-derived growth factor alpha; the PAI-1 encoding for plasminogen activator inhibitor type 1; the MET proto-oncogene, encoding for a membrane receptor protein with tyrosine–protein kinase activity, and others. The PDGF-A is expressed in primary and malignant melanoma and might function as an autocrine growth factor as well as an angiogenesis factor in tumor development. The PAI-1 is expressed in highly invasive metastatic human melanoma cell lines. The EGFR gene and the MET gene were independently amplified in human glioma. In malignant melanoma, the MET gene was shown to be expressed in metastatic lesions.

The permeability of this BBB is essentially regulated by TJ, the intercellular junction, in which the outer cell membranes are joined tightly together by rows of membrane proteins. TJ regulates the flow of ions, nutrients, and cells into the brain (Dejana, 2004; Abbott et al., 2006). The germ line mutation in genes involved in TJ regulation could disrupt BBB functioning. A number of recent studies strongly support the connection between melanoma metastasizing and TJ destabilization (Leotlela et al., 2007; Fazakas et al., 2011; Jayagopal et al., 2011). The important components of TJ are a family of proteins called “claudins.” Twenty-four such proteins are currently known. Genes CLDN2, CLDN3, CLDN4, CLDN11CL, CLDN12, CLDN14, and CLDN15 encoding for different claudins are also located on the chromosome 7 (Paperna et al., 1998; Hillier et al., 2003; Lal-Nag and Morin, 2009). One more TJ gene on chromosome 7 is OCLN, encoding for “occludin” protein. The localization of all these genes on one chromosome indicates that these genes together with other (not yet detected) genes on the same chromosome might represent an important part of the genetic mechanism linking the development of melanoma and brain metastases. If so, then performing association study of melanoma using SNP data from chromosome 7, and investigating functions of corresponding genes, may provide important insights about biological mechanisms connecting melanoma and brain metastases.

## Data from long life family study support the idea about role of pleiotropic genes in melanoma brain metastases

The Long Life Family Study (LLFS) involves four field centers (Boston, New York, and Pittsburg in the USA and Denmark in Europe). The recruitment and enrollment were conducted between 2006 and 2009. Potential probands were identified via Medicare enrollee, Danish Social Register lists, and articles appearing on the internet. Altogether, the LLFS enrolled 583 families with 1493 probands and their siblings and 192 spouses in the older generation, 2437 offspring and 809 of their spouses. The analyses did not include individuals with missing data on prevalence of melanoma or observed covariates. The remaining sample included 4638 individuals; 110 (2.4%) of them have prevalent melanoma. Among those, there were 2551 females [51 (2%) with melanoma] and 2087 males [59 (2.8%) with melanoma]. The tag SNPs for LLFS were produced by running HaploView with windows of size of 2000 markers at a time and *r*^2^ = 0.8. These markers were used in the LLFS principal component analyses (PCA). Tag SNPs were calculated for SNPs with MAF ≥ 5%; HWE *p*-value ≥ 1E-6.

The association study of this disease used LLFS data and tag SNPs located on chromosome 7. The prevalence of melanoma among the LLFS participants was considered as phenotype of interest. Note that according to SEER data the lifetime risk of melanoma is about 2%. Assuming that the genetic variant we are looking for is responsible for not more than 75% of melanoma cases and the lower boundary for a penetrance function corresponding to genetic variant associated with melanoma is not <0.1 the genetic frequency of the corresponding gene should not exceed 15%. The *p*-value threshold correcting for multiple testing was 1.7E-5. We used the EMMAX software package which allowed us to evaluate relatedness among family members using SNP data and take it into account in the analyses of family data (Kang et al., 2010). Observed covariates included gender, field center, generation (probands/offspring), and smoking habit (ever or never smoked). Twenty principal components were used to control for possible population stratification.

The analyses resulted in one genetic variant reaching chromosome-wide level of significance. The minor allele (T) of the rs208353 SNP was found to be associated with melanoma (*p* = 7.07E-6). Note that the estimate of MAF of this allele is about 7% which is in agreement with the assumption of MAF < 15% used in calculation of *p*-value threshold. The detected rs208353 SNP is located in the intron region of the GNA12 gene (synonyms: *GNA*12 | MGC104623 | MGC99644 | NNX3 | RMP | gep), which encodes guanine nucleotide binding protein (G protein) alpha 12. This finding supports recent result of Cardenas-Navia et al. (2010) who found that GNA12 and six other G-protein genes are frequently mutated in melanoma (somatic mutations). The literature review showed that the GNA12 gene plays a critical role in regulating TJ, which in turn is an essential component of the BBB permeability. The loss of endothelial TJ function was suggested to be an important event in the disruption of the BBB and promoting tumor metastases (Förster, 2008; Feng et al., 2011). The role of GNA12 is not limited to its involvement in melanoma and corresponding brain metastases. Several studies demonstrated the involvement of GNA12 in other cancers, potentially through compromised regulation of TJ and BBB permeability in carriers of some variants of this gene. Meyer et al. (2003) have shown that GNA12 directly affects Zona-Occludens proteins (ZO-1) and (ZO-2) which are usually localized at sites of intercellular junctions. It also interacts with the Src gene. ZO-1, ZO-2, and Src genes are involved in cancer growth and metastasis (Kaihara et al., 2003; Satomi et al., 2011; Creedon and Brunton, 2012). Sabath et al. (2008) have shown that TJ can be disrupted by GNA12-stimulated Src phosphorylation of ZO-1 and ZO-2 (TJP2). Kumar et al. (2006) and Kelly et al. (2006, 2007) demonstrated the ability of GNA12 to promote neoplastic transformations. Gan et al. (2011) showed that GNA12 is over-expressed in oral squamous cell carcinoma, and the over-expression drives migration and invasion of oral cancer cells. Juneja and Casey (2009) provided evidence that the G12 subfamily has been implicated in cancer cell invasion and metastasis. G12 signaling promotes prostate, breast, and ovarian cancer cell invasion *in vitro*, and these proteins are highly expressed in metastatic cancer tissues. Other genes that interact with GNA12 include tumor suppression gene TP53 and TJ gene TJ1. GNA12 also influences non-cancerous health disorders, such as ulcerative colitis and depression (Anderson et al., 2011; Lees et al., 2011; Zhang et al., 2012).

Thus, the GNA12 has pleiotropic health effects. Its germline variants have been significantly associated with melanoma in the LLFS data; it was also detected in an independent study of somatic mutations in melanoma (Cardenas-Navia et al., 2010); it is involved in TJ regulation important for permeability of BBB; and it plays role in many cancers as well as some other health disorders.

The variant from chromosome 7 next most significantly associated with melanoma in the LLFS data is the minor allele (T) of the rs55750236 SNP located in the KIAA1549 gene. Despite the fact that the *p*-value (*p* < 8.7E-5) of this analysis slightly exceeded the chromosome-wide significance level, this association is likely to be true positive. The KIAA1549 gene is known for its fusion with BRAF gene involved in the MAPK/ERKs signaling pathway which is thought to play a pivotal role in melanoma as well as other cancer development (Dahiya et al., 2012; Lin et al., 2012; Wangari-Talbot and Chen, 2012; Lewis et al., 2013). The KIAA1549-BRAF fusion itself was implicated in brain tumors (Badiali et al., 2012; Lin et al., 2012).

The analysis described above does not preclude association studies of melanoma using genetic variants located on other chromosomes. Several melanoma-related genes were found on other chromosomes, including G-protein-coupled receptors (e.g., GRM1) that are also involved in brain function (see, e.g., Wangari-Talbot and Chen, 2012). Thus, additional studies are needed to develop a more complete picture of genetic mechanisms connecting melanoma and brain metastases, as well as connecting the pathological effects of genes located on different chromosomes. The benefit of focusing on chromosome 7 is related to the specific research question addressed in this paper exploiting the fact that quite a number of genes involved in melanoma development and BBB regulation are located in this part of the genome. Another benefit deals with a smaller number of hypotheses testing in a genetic association study that substantially reduces the number of false positives compared to the genome wide association study dealing with SNPs from the entire genome. The important finding of this study is that mutation in the GNA12 gene can influence both the development of melanoma and the permeability of the BBB, and thereby contribute to the progression of melanoma to its metastatic state. The results of this paper also indicate the important role of genetic variants with pleiotropic effects in the developing of multiple health disorders. Recently, Jornsten et al. (2011) used data on glioblastoma available at The Cancer Genome Atlas (TCGA) to construct network models of mRNA expression. They found that the GNA12 gene is involved in network of disease-relevant hub genes that influence patient survival. The data on Skin Cutaneous Melanoma were just recently included into TCGA, so this resource can be used in the near future to validate roles of pleiotropic effects of genes in melanoma metastases. Targeting the pleiotropic genes could be an efficient strategy for simultaneous prevention and treatment of many health conditions.
